# Branched-chain amino acid transferase 2 (BCAT2) deficiency: A case series and systematic review

**DOI:** 10.1016/j.ymgmr.2026.101291

**Published:** 2026-01-17

**Authors:** Maja Filipic, Ziga Iztok Remec, Ana Drole Torkar, Nataša Sustar, Vanja Cuk, Chiara Rodaro, Maruša Debeljak, Matej Mlinaric, Jaka Sikonja, Vesna Bancic Silva, Primoz Kotnik, Tadej Battelino, Mojca Zerjav Tansek, Urh Groselj, Barbka Repic Lampret

**Affiliations:** a Faculty of Medicine, University of Ljubljana, Ljubljana, Slovenia; b Clinical Institute for Special Laboratory Diagnostics, University Children's Hospital, University Medical Centre Ljubljana, Ljubljana, Slovenia; c Department of Endocrinology, Diabetes, and Metabolism, University Children's Hospital, University Medical Centre Ljubljana, Ljubljana, Slovenia; d Department of Child, Adolescent and Developmental Neurology, University Children's Hospital, University Medical Centre Ljubljana, Ljubljana, Slovenia; e Clinical Department of Medical, Surgical and Health Science, University of Trieste, Trieste, Italy; f Department of Endocrinology, Diabetes, and Metabolic Diseases, Division of Internal Medicine, University Medical Centre Ljubljana, Ljubljana, Slovenia

**Keywords:** BCAT2, Branched-chain amino acids, Branched-chain amino acid transaminase 2, Hypervalinemia, Hyperleucine-isoleucinemia, Insulin resistance, White matter abnormalities

## Abstract

**Background:**

Branched-chain amino acid transaminase 2 (BCAT2) deficiency is an autosomal recessive disorder that impairs branched-chain amino acid (BCAA) catabolism. Its clinical and metabolic features remain poorly understood due to limited reports in the literature.

**Methods:**

We report three novel cases of BCAT2 deficiency from Slovenia: one diagnosed following symptom onset, one through cascade screening of parents, and one by newborn screening. Diagnosis was established through metabolic evaluation and confirmation of pathogenic variants in the *BCAT2* gene. In addition, we performed a systematic review of all previously reported cases of BCAT2 deficiency.

**Results:**

All three patients were homozygous for the NM_001190.4:c.600C > A (p.Tyr200Ter) variant, with valine concentrations at presentation of 2093, 2589, and 794 μmol/L. Only one patient was symptomatic, presenting with headaches, developmental delay, and intellectual disability, while the remaining two were largely asymptomatic. Notably, insulin resistance was observed in one of the three patients and may be associated with elevated BCAA levels. Systematic literature review identified 8 additional cases of BCAT2 deficiency. Genetic variant c.600C > A was also found in two Pakistani individuals, while the remaining variants were each reported in only a single individual. The most common clinical characteristics were intellectual disability (55%), developmental delay and other neurological symptoms (36%). Abnormal white matter findings on MRI were observed in all patients who underwent imaging. BCAA levels decreased in all patients receiving pyridoxine supplementation; however, only 50% showed clinical improvement.

**Conclusion:**

BCAT2 deficiency displays marked interindividual heterogeneity, ranging from asymptomatic cases to severe neurological impairment, which renders its pathogenicity uncertain.

## 1 Background

Branched-chain amino acids (BCAAs) – leucine, isoleucine and valine – are essential amino acids. Metabolically, they regulate protein synthesis and turnover, influence signaling pathways, and are involved in glucose homeostasis [1], [2].

**Fig. 1:**
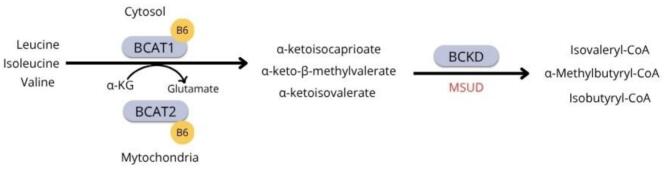
First two reactions of branched-chain amino acids catabolism. α-KG: α-ketoglutarate. BCAT: branched-chain amino acid transferase. BCKD: branched-chain keto acid dehydrogenase. MSUD: maple syrup urine disease.

BCAAs are broken down by transaminase and decarboxylase enzymes. Unlike most amino acids, only a small fraction is metabolized by the liver, while the majority enters circulation to reach muscles, adipose tissue, and the brain [3], [4]. The first two steps in the metabolism of BCAAs are common (Fig. 1): the initial step involves reversible transfer of the BCAA amino group to α-ketoglutarate, catalyzed by the mitochondrial (BCAT2) or cytosolic (BCAT1) isoforms of branched-chain amino acid transaminase (BCAT), resulting in the formation of glutamate and the corresponding branched-chain keto acids [5]. In the next step, the branched-chain keto acid dehydrogenase complex (BCKD) catalyzes oxidative decarboxylation [2]. Pyridoxal 5′phosphate, the active form of vitamin B6, is a quintessential coenzyme in the first step of BCAA metabolism, facilitating the transfer of the amino group when bound to BCAT [6].

Genetic defects in one of the two steps disrupt the catabolism of BCAAs and result in a substantial elevation of BCAAs in plasma, brain and other tissues. Maple syrup urine disease (MSUD) is caused by biallelic pathogenic variants in genes encoding the BCKD complex that leads to severe neurological dysfunction [7]. Growing evidence indicates that neurotoxicity in MSUD is primarily driven by the accumulation of branched-chain amino acids (BCAAs) and their corresponding branched-chain α-ketoacids (BCKAs), particularly leucine and α-ketoisocaproate. These metabolites impair neurotransmitter and cerebral protein synthesis, disrupt redox homeostasis, compromise mitochondrial energy production, and provoke an inflammatory response in the central nervous system [8].

In contrast, in BCAT deficiency only BCAAs are elevated, without a concomitant increase in BCKAs [9]; however, it remains unclear whether elevated BCAAs alone are sufficient to cause neurological dysfunction. BCAAs themselves can disrupt the transport of other amino acids across the blood–brain barrier and have been implicated in promoting a pro-oxidative state and driving inflammation [8]. In animal models, BCAT deficiency has been linked to elevated plasma and tissue BCAA levels, which were associated with hypertrophy of the heart, kidney, and spleen [10]. Moreover, increased BCAA levels and related metabolites have been identified as a “metabolic signature” of obesity, insulin resistance, and glucose intolerance, implicating altered BCAA metabolism in the regulation of glucose homeostasis [11].

To date, there are only eight reported cases with biallelic pathogenic variants in the *BCAT2* gene [9], [12], [13], [14], [15], reflecting either the exceptional rarity of this disorder or the possibility of a low-symptom or asymptomatic natural disease course, as well as gaps in our understanding of its possible pathophysiology. The aim of the following paper is to present three novel cases with BCAT2 deficiency from Slovenia and conduct a systematic literature review to consolidate the existing knowledge of all reported cases of this disorder.

## 2 Methods

### 2.1 Case series

We performed a retrospective evaluation by assessing electronic medical records of two pediatric patients who were followed at the University Children's Hospital, University Medical Centre Ljubljana up until the ages of 20.5 (Case 1) and 5.5 years (Case 3), and the mother of the former, who underwent genetic testing and clinical evaluation at the age of 39.2 years (Case 2). Written informed consent was obtained from the patients' parents/guardians for the inclusion into the study. CARE reporting guidelines were followed [16].

Diagnosis of the BCAT2 deficiency was established through the patient's metabolic profiles and confirmed with genetic testing. Results and clinical characteristics were compared to similar cases in the literature.

Plasma amino acids were quantified using MassChrom Amino Acid Analysis in Plasma/Serum kit (Chromsystems, Gräfelfing, Germany) on tandem mass spectrometer in MRM mode according to manufacturer MS method (Waters Xevo TQSmicro, Massachusetts, USA). Acylcarnitines were analyzed from dried blood spots (DBSs) on tandem mass spectrometer (Waters Xevo TQD, Massachusetts, USA) using NeoBase™ 2 Non-derivatized MSMS kit (PerkinElmer, Turku, Finland). Urine organic acids were measured with an in-house method on Agilent 5975C Series GC/MSD (Agilent Technologies, USA) on Agilent Ultra2 column (Agilent Technologies, USA).

In case 1, sequence analysis of the *BCAT2* gene (OMIM: 113530) was performed. The *BCAT2* gene was analyzed by PCR and Sanger sequencing, covering the entire coding region and the highly conserved exon-intron splice junctions, with the reference sequence *BCAT2*: NM_001190.4. Additionally, whole exome sequencing was conducted using next-generation sequencing (NGS) to clarify other clinical features. The NGS library was prepared with the Illumina DNA Prep. and enriched using the xGen Exome Research Panel v2 (IDT). A targeted interpretation was performed on genes associated with the clinical presentation of short stature, myopia, and obesity. More than 95% of the coding regions of the analyzed genes achieved sufficient sequencing coverage, with an average coverage of 81× for the regions analyzed. No clinically significant variants regarding short statue, myopia and obesity have been found.

For case 2, who is the mother of case 1, sequence analysis of the exon 6 of the *BCAT2* gene was performed. The *BCAT2* gene was analyzed by PCR and Sanger sequencing of both DNA strands.

Case 3 was discovered as part of expanded newborn screening with the sample collected in the second day of life, using NeoBase™ 2 Non-derivatized MSMS kit (PerkinElmer, Turku, Finland). Confirmatory DBS was taken on day 11, together with plasma for AA analysis, urine for organic acids analysis, and blood for isolation of DNA for NGS. Genetic analysis was performed using NGS. Regions of interest were enriched using the Agilent Sureselect^XT^ Target Enrichment System (Agilent Technologies, Inc., ZDA), followed by confirmatory testing with Sanger sequencing. A panel of 72 genes associated with inborn errors of metabolism, including the *BCAT2* gene, was used. In the analyzed genes, sequencing coverage reached 136× for the targeted regions. Targeted interpretation of the coding regions of the *BCAT2, DBT, BCKDHA, BCKDHB* and *DLD* genes was conducted, with more than 95% of the regions meeting the appropriate sequencing quality parameters.

Enzyme activity testing of BCAT2 was not performed.

The UK-WHO charts were employed for computing percentiles / *Z*-scores for anthropometric measurements. Standard methods were utilized to analyze laboratory measurements derived from blood and urine samples.

Magnetic resonance imaging (MRI) of the brain, electrocardiogram, MR elastography and heart ultrasonography were conducted and interpreted by specialists in the respective field.

To assess glucose homeostasis, an oral glucose tolerance test (OGTT) was performed. In cases 1 and 2, a standard dose of 75 g of glucose was used, whereas in Case 3 a lower dose of 1.75 g/kg body weight was administered due to a body weight below 43 kg. Glucose levels during the OGTT were interpreted according to the American Diabetes Association criteria for defining prediabetes and diabetes [17]. Hyperinsulinemia was defined as serum insulin levels exceeding 100 mU/L during the glucose load [18]. Insulin resistance was estimated using two widely accepted indices: the Matsuda Insulin Sensitivity Index and Homeostatic Model Assessment of Insulin Resistance (HOMA-IR) [18].

### 2.2 Systematic review of literature

**Fig. 2:**
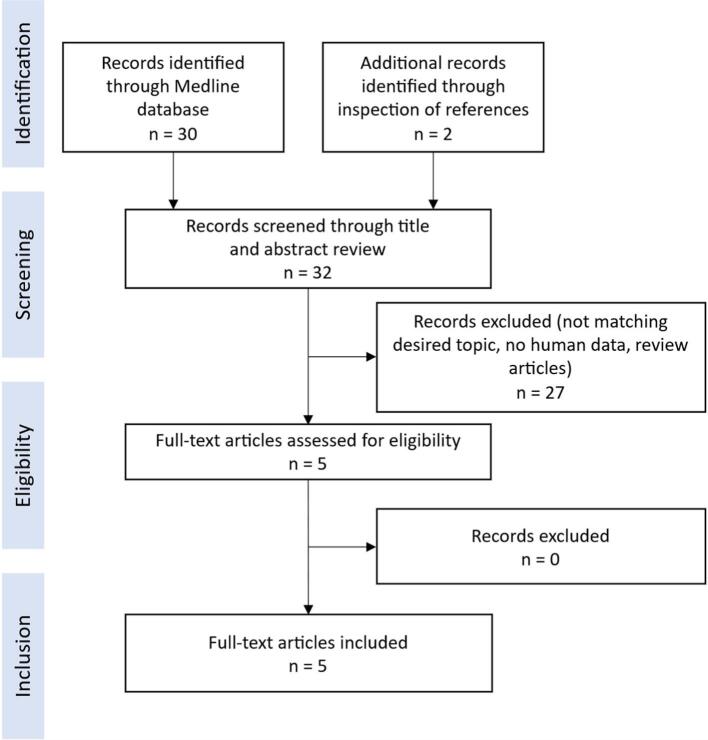
PRISMA flow diagram for systematic literature review.

A systematic literature review was performed following PRISMA reporting guidelines on May 12th 2025, to combine the data of all known patients with (likely) pathogenic variants in the *BCAT2* gene (Fig. 2). We searched the Medline database for available articles using the search terms “BCAT2”, “branched-chain aminotransferase 2”, “mutation”, “deficiency”, “BCAA”, “branched-chain amino acids”, “hypervalinemia” and “hypervaline-isoleucinemia” and identified 30 different research articles. Additionally, we reviewed the references of the identified articles and found two more articles.

During the screening of article titles and abstracts, we excluded in vitro studies, animal model studies, review articles, articles unrelated to the desired topic, and non-original case reports. The remaining 5 articles reporting original patient data were assessed thoroughly for eligibility and included in the systematic review.

## 3 Results

### 3.1 Case series

#### 3.1.1 Case presentation 1

Patient 1 is a 20-year-old female of Romani descent, referred to the Department of Endocrinology, Diabetes, and Metabolic Diseases University Children's Hospital Ljubljana at the age of 14 for evaluation of recurrent headaches, hirsutism, obesity, and irregular menstrual cycles, suggestive of an underlying metabolic disorder.

The patient is the first child of parents with no known consanguinity and has a healthy 4-year-old half-sister. Notably, three of her paternal uncles had severe sensory, cognitive and motor impairments. Labor was induced at 36 weeks gestation, with a birthweight of 2000 g, and phototherapy was required post-birth due to neonatal jaundice.

She began articulating intelligible words in her first year of life, but did not begin walking until the age of 20 months. Up to the age of 6 years, she had frequent hospitalizations for bronchitis. At 7 years old, she was also diagnosed with myopia, which progressively worsened. Menarche occurred at age 12, though her menstrual cycles remained irregular. She had a history of progressive learning difficulties, especially affecting memory, repeated several school years, and only completed primary education through an adapted program.

At the age of 12 years, she began experiencing recurrent headaches. There was no apparent impairment in hearing, speech, or motor function. At the age of 14 years, she was referred to a neurologist, and clinical examination revealed reduced muscle tone without asymmetry a short neck, along with mild motor incoordination, particularly in the right hand, and an unstable tandem gait. Her height was 151 cm, corresponding to the 6th percentile (P6), which was below her genetic potential. She was significantly overweight, with marked abdominal fat accumulation (body weight 70 kg, P97; body mass index [BMI] 30.7 kg/m^2^, P100).

She met the criteria for polycystic ovary syndrome (PCOS) in adolescence [19]: persistent menstrual irregularities and hyperandrogenism. Hyperandrogenism was present clinically, manifested by male-pattern hirsutism and persistent acne, and biochemically, with total testosterone in the high normal range (1.8 nmol/L; reference range [RF]: 0.1–2.0) and an increased free androgen index (FAI; 11.5; RF: <7). Ovarian ultrasound demonstrated polycystic ovarian morphology. Gonadotropin concentrations (FSH 6.1 U/L [RF: 1.7–18.5]; LH 13.4 U/L [RF: 0.6–69.9]), as well as thyroid hormone and prolactin levels, were within normal reference ranges, and alternative causes of hyperandrogenism were excluded.

A borderline prolonged QTc interval was observed on the electrocardiogram; however, the heart ultrasound revealed a structurally normal heart with normal function. MR elastography showed stage 2 liver fibrosis without focal lesions and mild fatty infiltration (3%). Ophthalmologic examination identified myopia, lens capsule opacities, and slightly flattened retinal vessels, with no evidence of leukodystrophy-associated vasculopathy.

**Fig. 3:**
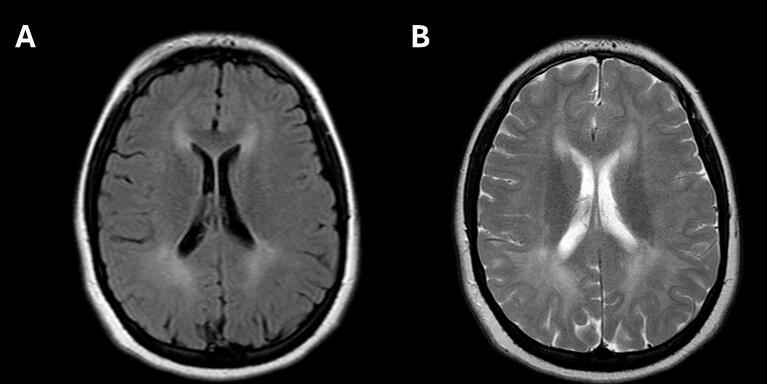
White matter abnormalities on magnetic resonance imaging: FLAIR sequence (A) and T2-weighted sequence (B).

At the age of 14 years, MRI of the head revealed a diffuse, markedly increased signal on FLAIR and T2-weighted sequences in the deep white matter, with lesser involvement of the subcortical white matter (Fig. 3). The signal abnormalities were evenly distributed across all brain lobes, with slightly lower intensity in the bilateral temporal regions. Signal characteristics of infratentorial structures were normal.

Metabolic studies at the age of 14 years showed markedly elevated plasma valine (2093 μmol/L; 6.5-times above upper limit of normal [ULN]), leucine (748 μmol/L; 3.5-times above ULN) and isoleucine (622 μmol/L; 5.8-times above ULN), but there was no elevation of allo-isoleucine and BCKAs in urine, concentrations of other amino acids in plasma were normal.

Laboratory results following a controlled fast showed elevated levels of pyruvate (122 μmol/L; RF: 34–102 μmol/L) and blood ammonia (51 μmol/L; RF: 9–33 μmol/L), along with an isolated elevation in ALT (1.48 μkat/L [88.8 IU/L]; RF: <0.56 μkat/L [<33.6 IU/L]).

A standardized 75 g 2-h oral glucose tolerance test (OGTT) was performed prior to the initiation of treatment at the age of 14.8 years. At that time, her body weight was 66.9 kg (P93), BMI 30.1 kg/m^2^ (P99) and HbA1c was 5.4%. OGTT demonstrated impaired glucose tolerance, with normal fasting glucose of 4.3 mmol/L (RF: <5.6 mmol/L) and an elevated 2-h post-load glucose level of 9.7 mmol/L (RF: <7.8 mmol/L). Fasting insulin was 21.0 mU/L (RF: 2–29.1 mU/L), while post-load insulin levels were markedly elevated, peaking at 1072.0 mU/L. Insulin resistance, assessed by the Matsuda Insulin Sensitivity Index (0.71) and HOMA-IR (4.0), was substantially more pronounced than that reported in a cohort of overweight Slovenian adolescents of comparable age [18].

Levels of lactate, folic acid and vitamin B12 were normal, and transferrin phenotyping showed a normal distribution. Evaluation for neurodegenerative disorders conducted at an external laboratory showed no abnormalities.

Genetic analysis of the *BCAT2* gene showed a homozygous variant NM_001190.4:c.600C > A (p.Tyr200Ter) that results in a premature stop codon. The variant was classified as likely pathogenic according to the American College of Medical Genetics and Genomics criteria.

Following diagnosis of BCAT2 deficiency, the patient was started on high-dose vitamin B6 (200 mg b.i.d.). A follow-up after one month showed a reduction in the plasma BCAA levels (18.3% for valine, 24.5% for leucine and 15.8% for isoleucine; see Table 2). At this follow-up visit, metformin (1000 mg b.i.d.) was prescribed to treat insulin resistance, and a reduction diet with sufficient physical activity was advised due to obesity. For the management of PCOS, the patient was initiated on combined oral contraceptives.

Following combined pharmacological and non-pharmacological treatment, her symptoms showed overall improvement. By the last follow-up at 20 years of age, her body weight had improved markedly, reaching 61.3 kg (P75) and a BMI of 27.1 kg/m^2^ (P94). Her muscle tone and tandem gait improved, and headaches became milder and less frequent. Her mother also reported improved concentration and study ability. She began seeking employment after completing primary education with an adapted program.

#### 3.1.2 Case presentation 2

Patient 2 is the mother of patient 1, who underwent biochemical and genetic testing at 39.2 years of age after her daughter was diagnosed with BCAT2 deficiency.

The patient reported no health concerns or complaints and was not taking any medication. The family history was unremarkable, and clinical examination revealed no abnormalities. Her body weight was 73 kg, corresponding to a BMI of 31.6 kg/m^2^. The analysis of plasma amino acids revealed significantly elevated BCAA levels: valine at 2589 μmol/L (7.7-times above ULN), isoleucine at 775 μmol/L (7.2-times above ULN), and leucine at 822 μmol/L (4.1-times above ULN). The acylcarnitine profile and organic acid analysis were within normal limits.

Further phenotypic characterization of the disorder with an OGTT revealed impaired glucose tolerance (fasting glucose 5.1 mmol/L [RF: <5.6], 2-h glucose 8.7 mmol/L [RF: <7.8]) and hyperinsulinemia (fasting insulin 5.0 mU/L, peak insulin 238 mU/L), while HbA1c was 5.2%, HOMA-IR was within normal range (1.02) and the Matsuda index (2.96; range for insulin resistance <2.5) indicated borderline insulin resistance. Laboratory analysis also showed an isolated elevation in the ALT (1.18 μkat/L; RF: <0.57 μkat/L), as well as a borderline lipid profile, with a slightly elevated total cholesterol (5.3 mmol/L; RF: <5.2 mmol/L), LDL-cholesterol (3.4 mmol/L; RF: <3.4 mmol/L) and triglycerides (3.3 mmol/L; RF: <1.7 mmol/L), and slightly lower HDL-cholesterol levels (1.2 mmol/L; RF: >1.3). The other tested parameters were within normal ranges.

Genetic testing confirmed the same homozygous variant in the exon 6 of the *BCAT2* gene as in her daughter (NM_001190.4:c.600C > A; p.Tyr200Ter).

The patient was started on metformin at a dose of 850 mg b.i.d. No follow-up visits were conducted.

#### 3.1.3 Case presentation 3

Our patient 3 is a 5-year-old Romani newborn, who was admitted to our department at 16 days of age due to increased BCAA levels in DBS detected through expanded newborn screening.

The patient's family history was unremarkable, and he had a healthy 3-year-old sister. The pregnancy was uncomplicated. He was born at 41 weeks gestation, with a birth weight of 3980 g, length of 53 cm and head circumference of 38 cm, all within normal range. Apgar score was 9/9. At birth, transient evoked otoacoustic emissions were absent bilaterally, and a follow up was scheduled.

During the first weeks of life his development was normal and no abnormalities were noted in the clinical examination. However, the results of the expanded newborn screening revealed elevations in the metabolites characteristic for MSUD in DBS: leucine+isoleucine (287 μmol/L and 561 μmol/L on days 5 and 11 of life, respectively; RF: <229 μmol/L) and valine (350 μmol/L and 531 μmol/L on days 5 and 11 of life, respectively; RF: <214 μmol/L).

Upon admission at 16 days of age, a further metabolic workup from a separate plasma sample confirmed elevations in all BCAA levels: valine at 794 μmol/L (4.2-times above ULN), leucine at 407 μmol/L (2.5-times above ULN), and isoleucine at 285 μmol/L (3.1× ULN). Allo-isoleucine was undetectable and BCKAs in urine were not elevated. Acylcarnitine profile was within normal limits.

Genetic analysis identified the same homozygous *BCAT2* variant as previously detected in patients 1 and 2 (NM_001190.4: c.600C > A; p.Tyr200Ter).

The patient was re-evaluated at 18 months of age. Parents reported that he began walking at 15 months and was developing normally, articulating several specific words. His appetite was normal, and he consumed a variety of foods without difficulty. Clinical examination showed no abnormalities, however, BCAA levels remained markedly elevated, with valine at 1755 μmol/L (6.0-times above ULN), leucine at 715 μmol/L (4.6-times above ULN), and isoleucine at 538 μmol/L (6.3-times above ULN). A neurological evaluation, carried out a month later, revealed no abnormalities, except for walking with a slightly wider base.

At last follow up at age 5.5 years, his weight was 29.5 kg (P100), height 120.7 cm (P91) and BMI 20.3 kg/m^2^ (P100). The physical exam showed no abnormalities, he was developing normally. The plasma aminoacids levels were as follows: valine at 1462 μmol/L (4.6-times above ULN), leucine at 654 μmol/L (3.0-times above ULN), isoleucine at 529 μmol/L (4.9-times above ULN). OGTT was performed during a short hospitalization after an overnight fast and revealed normal glucose homeostasis: fasting glucose 4.9 mmol/L (RF: <5.6 mmol/L), fasting insulin 2.9 mU/L (RF: 3–25 mU/L), post-load glucose 4.1 (RF: <7.8 mmol/L), post-load insulin 6.2 mUL/L, HbA1c 5.1%. Insulin resistance was absent based on a normal HOMA-IR (0.63) and Matsuda Index (13.55). Fasting lipid profile was normal.

No specific therapeutic measures were initiated due to limited cooperation of the patient's parents.

### 3.2 Systematic review of literature

We identified eight reported cases of BCAT2 deficiency in the literature. Table 1 summarizes the genetic background and clinical characteristics of eleven patients with *BCAT2* pathogenic variants, including eight previously reported cases and three from this study. Nine of eleven subjects (82%) were homozygous, the other two (18%) were compound heterozygous. Consanguinity was reported in three of nine (33%) patients with the homozygous genotype. Three patients (27%) were diagnosed through newborn screening, while the remaining cases were identified either through cascade screening of relatives or following symptom onset.

**Table 1:** List of all patients with BCAT2 deficiency reported in the literature and from this study with their most common clinical characteristics. Patients 1, 2, 3 from this study correspond to subjects IX, X and XI, respectively.

Subject	Reference	Age at diagnosis	Country of origin	*BCAT2* genotype	Hom / CH	Diagnosed through NBS	Clinical characteristics							
							Developmental delay	Neurological symptoms	Intellectual disability	Autism spectrum disorder	Recurrent headaches	Abnormal head MRI	Insulin resistance	Additional findings
I	Wang et al	25 years	China	c.509G > A; c.790G > A	CH	−	−	−	+	−	+	+	−	Steatohepatitis; Mildly impaired liver function; Moderate EEG abnormalities
II	Knerr et al	29 years	Italy	c.545 T > G; c.1021G > A	CH	−	+	NA	+	+	−	NA	−	
III	Knerr et al	11 years	Pakistan	c.600C > A	Hom	−	+	+	+	−	−	+	−	Microcephaly; Spastic paraparesis
IV	Knerr et al	37 years	Pakistan	c.600C > A	Hom	−	−	−	−	−	−	NA	−	
V	Knerr et al	4 years	Ireland	c.136_147del	Hom	−	+	−	+	+	−	+	−	
VI	Knerr et al., Navarrete et al., Martín-Rivada et al.	2 days	Spain	c.1154_1160delinsTGGATGCCCTCT	Hom	+	−	−	−	−	−	NA	−	
VII	Navarrete et al., Martín-Rivada et al.	2 days	Spain	c.1154_1160delins12	Hom	+	NA	NA	NA	NA	NA	NA	NA	
VIII	Mondésert et al	11 years	France	c.34C > T	Hom	−	−	+	+	−	−	+	−	Two acute neurological episodes with EEG abnormalities (facial paralysis; unilateral lower limb paresthesia; dysarthria)
IX	this study	14 years	Slovenia	c.600C > A	Hom	−	+	+	+	−	+	+	+	Obesity; PCOS; Myopia; Short stature; Borderline prolonged QTc interval; Mild fatty liver infiltration
X	this study	39 years	Slovenia	c.600C > A	Hom	−	−	−	−	−	−	NA	−	Dyslipidemia; Generally asymptomatic
XI	this study	16 days	Slovenia	c.600C > A	Hom	+	−	+	−	−	−	NA	−	Obesity; Generally asymptomatic
Proportion of patients						3/11	4/11	4/11	6/11	2/11	2/11	5/11	1/11	

Legend: Hom – homozygote. CH – compound heterozygote. NA – data not available. PCOS – polycystic ovary syndrome. NBS – newborn screening.

A total of nine unique pathogenic variants in the *BCAT2* gene were identified across the cohort. The most frequently reported variant, NM_001190.4: c.600C > A (p.Tyr200Ter), was found in all three individuals from the Slovenian cohort and in two patients from Pakistan, while the remaining variants were each reported in only a single individual. In the Slovenian cohort, patient 1 exhibited mild symptoms, while the other two patients were generally asymptomatic. Thus, among the five individuals carrying the most frequent variant (c.600C > A), three (60%) were largely asymptomatic.

The clinical presentation varied in severity across the cohort, with 5 of 11 individuals being asymptomatic. Although homozygous variants may increase the risk of a severe phenotype, five of nine (56%) homozygous individuals remained clinically asymptomatic, despite exhibiting an altered BCAA profile.

The most common clinical characteristics (Table 1) were abnormal brain MRI findings (5/5) and intellectual disability (6/11). These were followed by developmental delay and other neurological symptoms (4/11 each), autism spectrum disorder and recurrent headaches (2/11 each). Insulin resistance was the least frequent feature, documented in subject IX (our Case 1), with borderline insulin resistance additionally observed in subject X (our Case 2). Notably, brain MRI was not performed in all reported cases; however, abnormal findings were present in all patients who underwent head MRI.

The median concentrations of BCAAs at the time of diagnosis were 612.5 μmol/L (IQR: 387.25–766.5; *n* = 8) for leucine, 1528 μmol/L (IQR: 1106–2093; *n* = 9) for valine and 489.5 μmol/L (IQR: 348.75–660.25; n = 8) for isoleucine.

In 4/11 patients, oral pyridoxine supplementation was initiated as a potential treatment option, with the daily dosages ranging from 100 mg per day to 400 mg per day (our patient 1). In all the patients receiving pyridoxine supplementation, a reduction in the levels of BCAAs was observed, however, clinical symptoms improved in only 2 of the 4 patients, while the remaining 2 patients exhibited a stable clinical course without noticeable change (see Table 2).

**Table 2:** List of all patients with BCAT2 deficiency with their key laboratory findings before and after therapeutic intervention. Patients 1, 2, 3 from this study correspond to subjects IX, X and XI, respectively.

Subject	Reference	Age at diagnosis	*BCAT2* genotype	Homozygotes / CH	Stage at measurement	Leucine [μmol/L]	Valine [μmol/L]	Isoleucine [μmol/L]	Allo-isoleucine [μmol/L]	Clinical symptoms after treatment
I	Wang et al.	25 years	c.509G > A; c.790G > A	CH	Before treatment	Leu + Ile: 646	1755	NA	Not detected	Improvement after B6 supplementation
					After B6 treatment	Leu + Ile: 464 (28% reduction)	452 (74% reduction)	NA	Not detected	
II	Knerr et al	29 years	c.545 T > G; c.1021G > A	CH	No specific treatment	687	1528	505	<5	–
III	Knerr et al	11 years	c.600C > A	Hom	No specific treatment	328	1106	474	<5	No improvement after B6 supplementation
					After B6 treatment	NA	NA	NA	NA	
IV	Knerr et al	37 years	c.600C > A	Hom	No specific treatment	538	1108	370	<5	–
V	Knerr et al	17 years	c.136_147del	Hom	Before treatment	3446	3935	2774	<5	No improvement after B6 supplementation and protein-restricted diet
					3 months on diet (1.5 g natural protein/kg/day + BCAA-free mixture)	215 (94% reduction)	421 (89% reduction)	123 (96% reduction)	<5	
VI	Knerr et al., Navarrete et al., Martín-Rivada et al.	2 days	c.1154_1160delinsTGGATGCCCTCT	Hom	Before treatment	325	606	248	<5	Asymptomatic at 2.5 years, on a protein-restricted diet
					3 months on diet (1.8 g natural protein/kg/day + BCAA-free mixture)	86 (74% reduction)	173 (71% reduction)	61 (75% reduction)	NA	
VII	Navarrete et al., Martín-Rivada et al.	2 days	c.1154_1160delins12 (p.Ala385Valfs*35)	Hom	NA	NA	NA	NA	NA	–
VIII	Mondésert et al	11 years	c.34C > T	Hom	Before treatment	651	1158	514	2	No additional clinical episodes reported.
					After B6 treatment	462 (46.2% reduction)	984 (35.5% reduction)	302 (44.3% reduction)	NA	
IX	this study	14 years	c.600C > A	Hom	Before treatment	748	2093	622	<5	Improvement after B6 supplementation, metformin, oral contraceptives and nonpharmacological treatment of obesity
					After B6 treatment	565 (24% reduction)	1711 (18% reduction)	524 (16% reduction)	<5	
X	this study	39 years	c.600C > A	Hom	No specific treatment	822	2589	775	<5	–
XI	this study	16 days	c.600C > A	Hom	No specific treatment	407	794	285	<5	–
					18 months follow-up, no specific treatment	715	1755	538	<5	

Legend: Hom – homozygous; CH – compound heterozygous; NA – data not available.

Furthermore, two patients were started on a protein-restricted diet combined with supplementation of BCAA-free medical formulas, which resulted in a significant reduction in BCAA levels in both cases. One of them, who was discovered through expanded newborn screening, remained clinically asymptomatic at 2.5 years, while the other's clinical presentation was unchanged despite the significant improvement in laboratory values.

## 4 Discussion

Hypervalinemia and hyperleucine–isoleucinemia, in the absence of elevated urinary BCKAs and plasma allo-isoleucine, are hallmark biochemical features of BCAT2 deficiency – a disorder of BCAA catabolism described in only eight individuals to date. The clinical relevance of this condition remains uncertain, and its natural history is poorly understood. In this article, we report three additional cases of BCAT2 deficiency from two unrelated families, identified through different diagnostic approaches: one case was diagnosed following symptom onset, one through cascade screening of the parents of an index case, and one through newborn screening. The latter two individuals were asymptomatic or only mildly symptomatic despite marked elevations in plasma BCAAs, and all three individuals were homozygous for a nonsense variant in exon 6 of the *BCAT2* gene (NM_001190.4:c.600C > A; p.Tyr200Ter). In addition, we conducted a systematic literature review to update and consolidate the clinical and genetic characteristics of reported cases of BCAT2 deficiency.

Biallelic pathogenic variants in the *BCAT2* gene cause BCAT2 deficiency. Through the systematic review, nine unique pathogenic variants were identified. The most frequent variant, c.600C > A, was detected in all three newly reported cases in this study as well as in two previously reported cases from Pakistan, whereas each of the remaining variants has been described in only a single individual with BCAT2 deficiency. A possible explanation is that c.600C > A could represent a relatively common pathogenic variant in specific populations, with an overall allele frequency of 0.005% in the general population and slightly higher frequencies in the South Asian and European (non-Finnish) subpopulations according to gnomAD (aggregated data). Furthermore, all three newly reported cases were of Romani ethnic origin, and recent genome-wide ancestry analyses indicate that Roma derive from South Asia, most likely northwestern India—geographically near present-day Pakistan [20]. This shared ancestry may explain the occurrence of the same variant in our three cases and two cases from Pakistan. We propose that a founder effect and a high rate of consanguinity (although not reported in our cases) are the most likely explanations for the relatively high incidence of BCAT2 deficiency in the Slovenian Roma subpopulation [21]. A similar scenario has been observed in the Portuguese Roma, where a founder pathogenic variant in *BCKDHA* gene accounts for the high prevalence of MSUD in this community [22]. These findings have important clinical implications, as they enable the provision of carrier testing and prenatal diagnosis for at-risk families from the Roma ethnic group.

MSUD causes severe neurological impairment and, if untreated, can lead to early mortality [23]. In this condition, both BCAAs and BCKAs are elevated, with both believed to contribute to the neuropathology, although BCKAs are hypothesized to play a more prominent role [8]. In contrast, BCAT2 deficiency is a BCAA catabolic disorder, similar to MSUD, and is characterized by elevated BCAAs alone, and it remains unclear whether this elevation by itself causes any impairment.

There is considerable interindividual variability among patients with BCAT2 deficiency. Some individuals are largely asymptomatic (5/11 [45%]), while others exhibit modest to significant neurological impairment, despite significant elevations of plasma BCAAs in both groups. This variability may partly reflect differences in the method of detection. Individuals identified through newborn screening or cascade testing of relatives were generally asymptomatic or mildly affected, whereas those diagnosed after symptom onset presented with clinical manifestations. Notably, even patients with the same genotype, such as homozygotes for the c.600C > A variant, exhibit variable neurological impairment, with some individuals showing no detectable deficits. These observations highlight that the pathogenicity of BCAT2 deficiency remains largely uncertain, underscoring the need for further studies.

Abnormal brain MRI findings were observed in all patients (5/5) with BCAT2 deficiency who underwent imaging, whereas MRI was not performed in the remaining six patients. Unfortunately, it was not possible to distinguish between findings in symptomatic and asymptomatic patients, as all five individuals who underwent MRI were symptomatic. The reported changes appear to be consistent across cases [[9], [12], [13]], characterized by symmetrical, non-specific signal abnormalities: increased signal on FLAIR and T2-weighted sequences, and decreased signal on T1-weighted sequences. These abnormalities primarily involved the fronto-parietal white matter, occipital lobes, periventricular white matter, and corpus callosum, while the subcortical white matter and temporal lobes were less affected. No lesions were detected in the brainstem or cerebellum. White matter lesions remained stable over six months of vitamin B6 supplementation, despite a 35–45% reduction in plasma BCAA [9].

BCAAs serve as substrates for protein synthesis and energy production while also playing key roles in metabolic and signaling pathways, particularly through activation of the mammalian target of rapamycin (mTOR) pathway. Moreover, it has been suggested that hyperaminoacidemia, rather than hyperglycemia, drives insulin resistance and contributes to hyperinsulinemia in obesity [24]. Further supporting this idea, hyperaminoacidemia was found to enhance mTOR activity through an insulin-independent mechanism, directly affecting muscle tissue and further promoting insulin resistance [[25], [26]]. Based on this mechanistic rationale, one might expect insulin resistance and impaired glucose homeostasis to be prominent clinical features of BCAT2 deficiency. However, in our study, insulin resistance was clearly present in Case 1, borderline in Case 2, and absent in Case 3. Notably, in Cases 1 and 2, other, more common contributors to insulin resistance were observed, including obesity in both cases and PCOS in Case 1 [27]. Furthermore, insulin resistance has not been reported in any other previously published cases of BCAT2 deficiency. In summary, current evidence is insufficient to conclude that BCAA elevation resulting from BCAT2 deficiency directly causes insulin resistance or type 2 diabetes, despite a plausible mechanistic basis. Nonetheless, elevated BCAAs may potentially increase the risk of insulin resistance and type 2 diabetes through their effects on glucose metabolism and insulin signaling. Prospective studies and mechanistic research are required to validate this hypothesis and to better understand the causal pathways.

The main limitations of the present study include the relatively short duration of follow-up and the absence of radiological imaging data for the described patients 2 and 3, lack of comprehensive neuropsychiatric evaluation, and the lack of genetic and biochemical data from the parents of the included subjects. Although the reasons varied, the most prominent limitation arose from non-participation in clinical evaluation due to the personal beliefs of the patients or their parents.

## 5 Conclusions

This study broadens the current knowledge of BCAT2 deficiency by reporting three novel cases and integrating the available data from all known affected individuals. Individuals with BCAT2 deficiency exhibit considerable interindividual variability, ranging from largely asymptomatic to severe neurological impairment, despite comparable plasma BCAA elevations. Consequently, the pathogenicity of the disorder remains uncertain, and it is unclear whether BCAA elevation alone is sufficient to produce any impairment. A prominent feature in symptomatic individuals with BCAT2 deficiency is the presence of abnormal brain MRI findings, although it remains unclear whether such abnormalities are also present in asymptomatic individuals. The c.600C > A pathogenic variant is the most frequently observed among affected individuals and may constitute a population-specific common variant in groups such as the Slovenian Roma and Pakistani populations, potentially reflecting a founder effect and shared ancestry.

## Availability of data and materials

Raw data for dataset is not publicly available to preserve individuals' privacy under the European General Data Protection Regulation. The datasets used during the current study are available from the corresponding author on reasonable request.

## Originality of content

We confirm all information and materials in the manuscript are original.

## CRediT authorship contribution statement

**Maja Filipic:** Data curation, Conceptualization. **Ziga Iztok Remec:** Writing – review & editing, Data curation, Conceptualization. **Ana Drole Torkar:** Writing – review & editing, Data curation, Conceptualization. **Nataša Sustar:** Writing – review & editing, Data curation, Conceptualization. **Vanja Cuk:** Writing – review & editing, Conceptualization. **Chiara Rodaro:** Writing – review & editing, Data curation, Conceptualization. **Maruša Debeljak:** Writing – review & editing, Data curation, Conceptualization. **Matej Mlinaric:** Writing – review & editing, Data curation, Conceptualization. **Jaka Sikonja:** Writing – review & editing, Conceptualization. **Vesna Bancic Silva:** Writing – review & editing, Writing – original draft, Data curation, Conceptualization. **Primoz Kotnik:** Writing – review & editing, Conceptualization. **Tadej Battelino:** Writing – review & editing, Conceptualization. **Mojca Zerjav Tansek:** Writing – review & editing, Conceptualization. **Urh Groselj:** Writing – review & editing, Writing – original draft, Data curation, Conceptualization. **Barbka Repic Lampret:** Writing – review & editing, Writing – original draft, Formal analysis, Conceptualization.

## Consent for publication

Consent for publication was obtained from the parents of the individuals featured in the manuscript using our institutional consent form.

## Ethics approval and consent to participate

The study was conducted in accordance with the Declaration of Helsinki. The study and this paper were approved by the Scientific Committee of the Department of Endocrinology, Diabetes, and Metabolic Diseases, University Children's Hospital, University Medical Centre Ljubljana. Written informed consent has been obtained from the patient's parents to publish this paper and to results from genetic studies in an anonymized form.

## Funding

This work was partly supported by the Slovenian Research and Innovation Agency (grant P3-0343). The funding organization had no role in: design and conduct of the study; collection, management, analysis, and interpretation of the data; preparation, review, or approval of the manuscript; and decision to submit the manuscript for publication.

## Declaration of competing interest

The authors declare no competing interests.

## Data Availability

Data will be made available on request.

## References

[1] Monirujjaman Md, Ferdouse A. (2014). Metabolic and physiological roles of branched-chain amino acids. Adv. Mol. Biol..

[2] Lu J., Xie G., Jia W., Jia W. (2013). Insulin resistance and the metabolism of branched-chain amino acids. Front. Med..

[3] Fernstrom J.D. (2005). Branched-chain amino acids and brain function. J. Nutr..

[4] Hagenfeldt L., Eriksson S., Wahren J. (1980). Influence of leucine on arterial concentrations and regional exchange of amino acids in healthy subjects. Clin. Sci. Lond. Engl..

[5] Holeček M. (2018). Branched-chain amino acids in health and disease: metabolism, alterations in blood plasma, and as supplements. Nutr. Metab..

[6] Mann G., Mora S., Madu G., Adegoke O.A.J. (2021). Branched-chain amino acids: catabolism in skeletal muscle and implications for muscle and whole-body metabolism. Front. Physiol..

[7] Scharre S., Mengler K., Schnabel E., Kuseyri Hübschmann O., Tuncel A.T., Hoffmann G.F., idr. (2025). Impact of early diagnosis, disease variant, and quality of care on the neurocognitive outcome in maple syrup urine disease: a meta-analysis. Genet. Med..

[8] Amaral A.U., Wajner M. (2022). Pathophysiology of maple syrup urine disease: focus on the neurotoxic role of the accumulated branched-chain amino acids and branched-chain α-keto acids. Neurochem. Int..

[9] Mondésert E., Bouchereau J., Schiff M., Benoist J.F., Barcia G., Keren B., idr. (2025). Branched-chain amino acid transferase type 2 (BCAT2) deficiency: report of an eighth case and literature review. Mol. Genet. Metab. Rep..

[10] Zhenyukh O., Civantos E., Ruiz-Ortega M., Sánchez M.S., Vázquez C., Peiró C., idr. (2017). High concentration of branched-chain amino acids promotes oxidative stress, inflammation and migration of human peripheral blood mononuclear cells via mTORC1 activation. Free Radic. Biol. Med..

[11] Newgard C.B., An J., Bain J.R., Muehlbauer M.J., Stevens R.D., Lien L.F., idr. (2009). A branched-chain amino acid-related metabolic signature that differentiates obese and lean humans and contributes to insulin resistance. Cell Metab..

[12] Wang X.L., Li C.J., Xing Y., Yang Y.H., Jia J.P. (2015). Hypervalinemia and hyperleucine-isoleucinemia caused by mutations in the branched-chain-amino-acid aminotransferase gene. J. Inherit. Metab. Dis..

[13] Knerr I., Colombo R., Urquhart J., Morais A., Merinero B., Oyarzabal A., idr. (2019). Expanding the genetic and phenotypic spectrum of branched-chain amino acid transferase 2 deficiency. J. Inherit. Metab. Dis..

[14] Navarrete R., Leal F., Vega A.I., Morais-López A., Garcia-Silva M.T., Martín-Hernández E., idr. (2019). Value of genetic analysis for confirming inborn errors of metabolism detected through the Spanish neonatal screening program. Eur. J. Hum. Genet..

[15] Martín-Rivada Á., Palomino Pérez L., Ruiz-Sala P., Navarrete R., Cambra Conejero A., Quijada Fraile P., idr. (2022). Diagnosis of inborn errors of metabolism within the expanded newborn screening in the Madrid region. JIMD Rep..

[16] Riley D.S., Barber M.S., Kienle G.S., Aronson J.K., Von Schoen-Angerer T., Tugwell P., idr. (2017). CARE guidelines for case reports: explanation and elaboration document. J. Clin. Epidemiol..

[17] American Diabetes Association pProfessional pPractice Committee for Diabetes (2026 Jan 1). 2. Diagnosis and classification of diabetes: standards of care in diabetes-2026. Diabetes Care.

[18] Groselj U., Kafol J., Sikonja J., Mlinaric M., Sket R., Remec Z.I., idr. (2025). Enhanced oral glucose tolerance test for early detection of insulin resistance and metabolic complications in children with obesity. Am. J. Prev. Cardiol..

[19] Peña A.S., Witchel S.F., Boivin J., Burgert T.S., Ee C., Hoeger K.M., idr. (2025). International evidence-based recommendations for polycystic ovary syndrome in adolescents. BMC Med..

[20] Moorjani P., Patterson N., Loh P.R., Lipson M., Kisfali P., Melegh B.I. (2013). Reconstructing Roma history from genome-wide data. PLoS One.

[21] Quinn S., Walsh N., Streata I., Ververi A., Kulshrestha S., Puri R.D., idr. (2025). Catalogue of inherited autosomal recessive disorders found amongst the Roma population of Europe. Eur. J. Med. Genet..

[22] Quental S., Gusmão A., Rodríguez-Pombo P., Ugarte M., Vilarinho L., Amorim A., idr. (2009). Revisiting MSUD in Portuguese gypsies: evidence for a founder mutation and for a mutational hotspot within the BCKDHA gene. Ann. Hum. Genet..

[23] Hassan S.A., Gupta V. (2025 Jan.). StatPearls [Internet].

[24] Felig P., Marliss E., Cahill G.F. (1969). Plasma amino acid levels and insulin secretion in obesity. N. Engl. J. Med..

[25] Um S.H., D’Alessio D., Thomas G. (2006). Nutrient overload, insulin resistance, and ribosomal protein S6 kinase 1, S6K1. Cell Metab..

[26] She P., Olson K.C., Kadota Y., Inukai A., Shimomura Y., Hoppel C.L., idr. (2013). Leucine and protein metabolism in obese Zucker rats. PloS One.

[27] Herman R., Sikonja J., Jensterle M., Janez A., Dolzan V. (2023). Insulin metabolism in polycystic ovary syndrome: secretion, signaling, and clearance. Int. J. Mol. Sci..

